# Das „impulsive Irresein“ nach Emil Kraepelin: Ein Krankheitsbild für die Verbrecherinnen am Beginn des 20. Jahrhunderts

**DOI:** 10.1007/s00115-022-01286-2

**Published:** 2022-05-12

**Authors:** Teresa Rendel, Holger Steinberg

**Affiliations:** Forschungsstelle für die Geschichte der Psychiatrie, Klinik und Poliklinik für Psychiatrie und Psychotherapie, Medizinische Fakultät, Universität Leipzig, Semmelweisstr. 10, 04103 Leipzig, Deutschland

**Keywords:** Impulskontrollstörungen, Forensische Psychiatrie, Geschichte der Medizin, Frauengesundheit, Verbrechen, Impulse control disorders, Forensic psychiatry, History of medicine, Women’s health, Criminal behaviour

## Abstract

**Hintergrund:**

Als Teil seines klassifikatorisch-konzeptuellen Gesamtentwurfs beschreibt Emil Kraepelin zu Beginn des 20. Jahrhunderts detailliert das bis dahin wenig umgrenzbare Krankheitsbild des „impulsiven Irreseins“. Die dargestellten Erscheinungsformen entsprechen weitestgehend den als typisch weiblich bezeichneten Straftaten des späten 19. und frühen 20. Jahrhunderts.

**Fragestellung:**

Wie legt Kraepelin klassifikatorisch das „impulsive Irresein“ an und welche Formen beschreibt er? Trifft es zu, dass Kraepelin diese Störungen vorwiegend bei Frauen sieht, stellt er eine Verbindung zur Kriminalität von Frauen her und wie passt dies in die Diskurse der Zeit über Weiblichkeit, Strafrecht und Degeneration?

**Material und Methoden:**

Grundlage der Studie ist das von Emil Kraepelin beschriebene Krankheitsbild „impulsives Irresein“, wie er es in seinem Hauptwerk, der zwischen 1909 und 1915 erschienenen 8. Auflage seines *Lehrbuches der Psychiatrie*, niederlegt. Diese Ausführungen werden ausführlich analysiert und anhand von Sekundärliteratur in einen inhaltlichen und historischen Kontext gesetzt.

**Ergebnisse:**

In Rudimenten ist Kraepelins klinische Einteilung bis heute nachvollziehbar, wenngleich sich große inhaltliche Differenzen zu späterer Literatur zeigen. Kraepelin beschreibt das „impulsive Irresein“ eindeutig als mit dem Weiblichen besetzte Triebstörung.

**Diskussion:**

Mithilfe des „impulsiven Irreseins“ positioniert sich Kraepelin zu wesentlichen wissenschaftlichen Diskursen des frühen 20. Jahrhunderts wie der Strafrechtsdebatte und der Degenerationslehre. Anhand der einzelnen von Kraepelin beschriebenen Formen des „impulsiven Irreseins“ lassen sich verschiedene Konzepte zur Konstruktion und Pathologisierung von Weiblichkeit nachvollziehen. Es diente offenbar auch dazu, gängige Frauenverbrechen innerhalb der patriarchalen Hegemonie erklärbar zu machen.

## Abstract (en)

**Background:**

In his comprehensive classification of the beginning of the twentieth century, Emil Kraepelin provided a detailed description of an entity he called “impulsive insanity”, which had not been elaborated before him. The forms depicted by him largely corresponded to the offences, which were referred to as typically female in their nature in the late nineteenth and early twentieth centuries.

**Question:**

How did Kraepelin classify “impulsive insanity” and what forms did he describe? Did Kraepelin also see these disorders predominantly prevailing in women, did he establish a connection with women’s criminality and how did this fit into the discourses of the time on femininity, criminal legislation and degeneration?

**Material and methods:**

This study focused on the clinical picture “impulsive insanity” as described by Emil Kraepelin in his main work, the 8th edition of his *Textbook of Psychiatry* published between 1909 and 1915. His description was analyzed in detail and embedded in a historical context on the basis of secondary literature.

**Results:**

In rudiments Kraepelin’s clinical classification is still comprehensible today, although there are major differences to how literature in later years treated this issue. Kraepelin clearly sees “impulsive insanity” as a driving disorder predominantly prevailing in women.

**Discussion:**

Elaborating his concept of “impulsive insanity”, Kraepelin positioned himself in relation to important scientific discourses of the early twentieth century, such as the debate on criminal legislation and the theory of degeneration. On the basis of the individual forms of “impulsive insanity” described by Kraepelin, various concepts of constructing and pathologizing femininity can be identified. Apparently, it also aims to explain common female crimes within the patriarchal hegemony.

Was trieb so viele Frauen im 19. Jahrhundert dazu, gewisse Arten von Verbrechen zu begehen? Emil Kraepelin beleuchtet das Phänomen der Frauenverbrechen mithilfe des Krankheitsbildes „impulsives Irresein“, das er in seine wirkmächtige Nosologie psychischer Erkrankungen aufnimmt. Dabei berührt Kraepelin verschiedene wissenschaftliche Diskurse seiner Zeit, in denen unsere Studie ihn mithilfe seiner detaillierten Beschreibung dieser selten in den Blick genommenen Störung orientierend verorten möchte.

## Methodik

Als Ausgangspunkt unserer Studie wählten wir die 8. Auflage von Kraepelins Psychiatrielehrbuch, das in 4 Bänden zwischen 1909 und 1915 erschien. Seinen nosologischen Gesamtentwurf legte Kraepelin stets in den einzelnen Auflagen nieder. Da der Band zur allgemeinen Psychiatrie der letzten, der 9. Lehrbuchauflage von seinem Schüler Johannes Lange bearbeitet wurde und Kraepelin während der Arbeit am ersten Teil zur klinischen Psychiatrie verstarb und weitere Teile nie erschienen, kann die 8. Auflage als Endpunkt der detaillierten Darstellung seines krankheitskonzeptionellen Werkes gelten [25]. Sie wurde sogar als „bible of modern psychiatry“ bezeichnet [1, 23]. Das hierin als „impulsives Irresein“ beschriebene Krankheitsbild wurde sorgfältig inhaltlich analysiert und anhand von Sekundärliteratur sowohl in einen historischen als auch gegenwärtigen Kontext gesetzt.

## Emil Kraepelins Hauptleistung

Der 1856 in Neustrelitz geborene und 1926 in München verstorbene Emil Kraepelin gilt als Begründer der empirisch-klinischen Psychiatrie [15, 36]. Er besetzte psychiatrische Lehrstühle an den Universitäten Dorpat, Heidelberg und München, wo er zudem die Deutsche Forschungsanstalt für Psychiatrie im Wesentlichen begründete. Als strenger Vertreter einer undogmatischen, erkenntnistheoretischen Forschung [14] begriff er seine eigenen Theorien nicht als universale Gesetze, sondern stellte sie als heuristische Forschungsgrundlage zur Diskussion. Sein multikausales Krankheitsverständnis unter Zuhilfenahme verschiedener Disziplinen wie Empirie und Statistik, Psychopathologie, Längsschnittbetrachtung und Vererbungslehre änderte die Sicht auf psychische Erkrankungen maßgeblich und führte zur Überwindung der einseitigen Hirnanatomie und -histologie in der Psychiatrie, wenngleich er auch diese mit einbezog. Seine nicht – wie bis dato üblich – auf das klinische Symptombild versteifte, sondern als Gesamtkonzept empirisch-klinische Sicht, die insbesondere Krankheitsverlauf und Prognose als nosologische Parameter aufnahm, zeitigte den von ihm vor allem in seinem Kompendium niedergelegten Fortschritt psychiatrischer Klassifikation [14, 21]. In den Jahren um 1900 verfasste er als einer der Ersten überhaupt eine umfassende und schlüssige Nosologie psychischer Erkrankungen, die noch dazu von den Kollegen weitestgehend akzeptiert wurde, schnell Verbreitung fand und bis heute in ihren Grundbausteinen Bestand hat [15, 20, 33]. Die breite Anerkennung seines neuen Krankheitsverständnisses und seiner neuen Arbeitsweise, als deren Frucht seine Krankheitsklassifikation zu sehen ist, unterscheiden ihn dabei von anderen wichtigen französisch- und deutschsprachigen Nosologen des 19. Jahrhunderts. Dennoch war seine Forschung eine rein naturwissenschaftliche, soziale und biografische Krankheitsdimensionen interessierten ihn weit weniger. So lehnte er beispielsweise die Psychoanalyse als unwissenschaftlich ab. Wie sich in unserem Zusammenhang zeigen wird, ist es zudem bedeutsam, dass eines von Kraepelins frühesten Forschungs- und Interessensgebieten die Kriminalpsychologie war [4].

## Das Krankheitsbild des „impulsiven Irreseins“ nach Kraepelin

Kraepelin ordnet das „impulsive Irresein“ den Erscheinungsformen der „originären Krankheitszustände“ zu, so wie auch „die Nervosität“, „die Zwangsneurose“ und „die geschlechtlichen Verirrungen“. Insbesondere zu Letzteren sieht er die nächste Verwandtschaft. Das Krankheitsbild sei gekennzeichnet durch das Hervortreten einzelner, krankhafter Triebhandlungen, die ungeplant und gegen bessere Überzeugung ausgeführt würden. Der Trieb werde dabei im Gegensatz zu Zwangshandlungen als Ausdruck des eigenen Willens, also als icheigen erlebt, die Ausführung sei in der Regel mit einem starken Gefühl der Befriedigung und dem Abbau der Triebspannung verbunden. Bei dem „impulsiven Irresein“, das er innerhalb seiner Formenlehre *Klinische Psychiatrie* im IV. Lehrbuchband abhandelt, handle es sich um eine Form des „Entartungsirreseins“, es sei also Ausdruck eines ganzheitlichen psychischen Degenerationszustandes. Die Triebhandlung könne dabei viele Formen annehmen. Spezifiziert werden von Kraepelin diejenigen Formen, die eindeutig auf einen krankhaften Zustand schließen ließen [23].

### Die Formen des „impulsiven Irreseins“

Die impulsive Brandstiftung (Pyromanie) sei zum größten Teil eine Erkrankung unerfahrener junger Frauen, welche durch geringe Intelligenz, Alkoholismus der Eltern oder hysterische Veranlagung prädisponiert seien und in fremde Abhängigkeitsverhältnisse versetzt würden, denen sie zu entfliehen versuchten. Der impulsive Akt trete während der Menses auf. Die Erkrankung sei nach vollendeter Pubertät meist spontan regredient und habe daher eine gute Prognose, durch Strafen sei sie hingegen kaum zu beeinflussen.

Ähnlich verhalte es sich mit der impulsiven Kindstötung, womit hier explizit das Töten fremder Kinder bezeichnet wird, mit deren Betreuung die betreffende Person beauftragt wurde. Diese Form der Impulsstörung sei ausschließlich eine Erkrankung junger Frauen, wobei Kraepelin darauf hinweist, dass dieser Aspekt dadurch verursacht sein könne, dass beinahe ausschließlich Dienstmädchen angestellt würden. Auch hier läge eine Entwicklungsverzögerung mit geistiger Unreife vor, welche in Verbindung mit Heimweh, fehlender Supervision und Ausweglosigkeit aus einem erdrückenden Beschäftigungsverhältnis zur Ausführung des triebhaften Aktes führe. Aufgrund fehlender Reue sei die Wiederholungsgefahr hoch, hingegen sei auch hier mit dem spontanen Verschwinden des Triebes mit zunehmendem Alter zu rechnen.

Gänzlich anders ist hingegen die Typisierung der berechnenden, selbstsüchtigen und nach außen die perfekte Frau schauspielenden Giftmörderin. „Wenn die jungen Brandstifterinnen und Kindsmörderinnen Befreiung aus einer für sie unerträglichen Lebenslage suchen, […] entspringt hier das Handeln anscheinend aus der brennenden Begierde, Herr über Leben und Tod zu sein“ ([23], IV. Bd., S. 1906). Eng damit verwandt seien die anonymen Briefeschreiberinnen, die also fast immer weiblich seien und Freude daran empfänden, mit wenig Aufwand durch heimtückische Methoden Ärger und Verwirrung zu stiften. Nicht jeder Giftmord sei als triebhaft misszuverstehen, finanzielle Motive oder anderweitig materielle Interessen sprächen daher gegen diese Diagnose.

Der Kleptomanie, insbesondere dem triebhaften Warenhausdiebstahl, sei die Habsucht hingegen inhärent. Diese Form der Triebstörung sei eine Verstärkung normal vorhandener Gelüste. Die ansonsten unbescholtenen Frauen seien insbesondere während der Menses nicht mehr in der Lage, ihren Drang zu kontrollieren. Der Diebstahl könne mit geschlechtlicher Erregung oder orgastischer Befriedigung verbunden sein, entweder durch den Akt selbst oder durch das Stehlen eines fetischisierten Gegenstandes. Häufig seien hysterische Begleiterscheinungen zu beobachten.

Diese ließen sich vereinzelt auch beim triebhaften Schuldenmachen beobachten. Seine dahingehend diagnostizierten Patientinnen, allesamt weiblich, charakterisiert Kraepelin als naiv und vertrauensselig. Durch ihr mangelndes Verständnis für Finanzen ließen sie sich leicht von Gläubigern ausnutzen, was sie in eine Schuldenspirale triebe. Trotz der großen Ausgaben lebten sie sparsam. (alles in: [23], IV. Bd., S. 1901–1916).

## Impulskontrollstörungen im Wandel

Zum Zeitpunkt der Veröffentlichung seines Werkes, 1915, kam Kraepelin zu dem Schluss, dass impulsive Störungen wenig erforscht und schlecht eingrenzbar seien. Diese Einschätzung ist weiterhin zutreffend, denn trotz ihrer klinisch-psychiatrischen Relevanz gelten sie als noch immer vergleichsweise wenig erforscht [32]. Dennoch lassen sich impulsive Triebstörungen bis in Schriften der frühen Psychiatrie des 19. Jahrhunderts zurückverfolgen, und auch im Laufe des 20. Jahrhunderts bis heute scheint zumindest über deren grundsätzliches Vorhandensein weitestgehend wissenschaftlicher Konsens zu herrschen. Welche Formen der Triebhandlung für diese Erkrankung paradigmatisch seien und wie sie von anderen Erkrankungen mit Triebsteigerung abzugrenzen sei, unterliegt jedoch einem stetigen Wandel.

Als eine der ersten Erwähnungen einer dezidiert impulsiven Erkrankung gilt die „folie impulsive“ des französischen Psychiaters Jacques Joseph Valentin Magnan von 1892. Der bis heute als ein Schöpfer der medizinischen Degenerationslehre bekannt gebliebene Nervenarzt beschreibt hier einen immer stärker werdenden, immer schwerer zu unterdrückenden Impuls, der sich beispielsweise in suizidalen Tendenzen, Phobien und epileptischen Anfällen äußere [3]. Auch in den psychiatrischen Lehrbüchern des deutschsprachigen Raumes werden schon früh Erkrankungen erwähnt, die mit pathologischen Trieben wie Stehlen, Brandstiftung oder Kindstötung einhergehen, allerdings werden sie unterschiedlichen Entitäten zugeordnet. Wilhelm Griesinger wertet 1845 den Drang zu Mord und Zerstörung, namentlich das Brandstiften und Kindstöten, als Ausformung der Melancholie, weitere impulsive Akte hingegen als maniakalisch, wie die Fresssucht, Stehlsucht und gesteigerten Sexualtrieb [9], ebenso wie Richard von Krafft-Ebing, nach dessen Einschätzung impulsive Akte wie Mord, Stehlsucht oder Brandstiftung unter anderem durch maniakalische Zustände verursacht würden [24]. Karl Jaspers befasst sich in seiner Dissertation 1909 zudem intensiver mit der Verbindung von Heimweh und Triebverbrechen, insbesondere bei Dienstmädchen [17]. Im Gegensatz zu vielen seiner intellektuellen Vorgänger sieht Kraepelin gesteigerten Sexualtrieb nicht als eigene Triebstörung [31].

Aktuell werden sowohl die Pyromanie als auch Kleptomanie von den beiden wichtigsten internationalen Klassifikationssystemen psychischer Erkrankungen, dem ICD-11 (International Statistical Classification of Diseases and Related Health Problems 11) sowie DSM‑5 (Diagnostic and Statistical Manual of Mental Disorders 5), als Formen der Impulskontrollstörung durch einen eigenen Diagnostikcode anerkannt. Das DSM‑5 nennt zudem Störungen des Sozialverhaltens, oppositionelle Störung, antisoziale Persönlichkeitsstörung und intermittierend explosive Störung als weitere spezifische Formen dieser Impulserkrankung [2]. Im ICD-11 hingegen werden neben der intermittierend explosiven Störung noch Spielsucht, körperbezogene Impulskontrollstörungen wie Trichotillomanie und sog. Skin-Picking sowie impulsives Sexualverhalten angeführt [39], wobei Letzteres nur begrenzt mit der mittlerweile obsoleten Diagnose Nymphomanie gleichzusetzen ist [31]. Deutschsprachige psychiatrische Standardlehrwerke orientieren sich eher an der ICD-Klassifikation.

Laut DSM‑5 sei die genaue Prävalenz der Pyromanie als sehr seltene Primärdiagnose nicht bekannt. Im Gegensatz zu Kraepelins Einschätzung seien Männer weit häufiger betroffen. Hingegen deckt sich mit ihm die dortige Angabe über das Geschlechterverhältnis der Kleptomanie, deren Prävalenz bei 0,3–0,6 % der Bevölkerung liege und von welcher Frauen etwa dreimal so oft betroffen seien [2].

Neuere englischsprachige Veröffentlichungen zum Thema Pyromanie betonen, dass die Erkrankung selbst unter Brandstiftenden eine weit geringere Prävalenz habe als bisher angenommen und insgesamt extrem selten sei. Unter diesen seltenen Fällen sei eine Verbindung mit sexueller Befriedigung noch seltener [5, 18]. Noch 1997 betonte der Psychiater Hartmann Hinterhuber hingegen besonders die sexuelle Komponente beim triebhaften Stehlen und Brandstiften [13].

Kindstötung, Giftmischen und Schuldenmachen haben seit Kraepelins Veröffentlichung als Impulskontrollstörung keine Relevanz mehr.

## Bezugnahme auf wissenschaftliche Diskurse des 19. Jahrhunderts

Die Psychiatrie ist stärker als jeder andere medizinische Fachbereich durch gesellschaftliche Entwicklungen geprägt und beeinflusst diese wiederum selbst. Zugleich kämpfen zahlreiche philosophische und naturwissenschaftliche Konzepte um die Vorherrschaft in der Auffassung von der menschlichen Psyche. Mit der Ausformulierung des „impulsiven Irreseins“ positioniert Kraepelin sich zu zwei wesentlichen Diskursen der Jahrhundertwende, die helfen, sein Weltbild nachzuvollziehen: dem theoretisch-ätiologischen Konzept der Degenerationslehre sowie der forensisch-psychiatrischen Strafrechtsdebatte.

### Degenerationslehre statt Monomanie

Kraepelin hing wie viele Nervenärzte seiner Zeit der umstrittenen Degenerationslehre an, einem medizinischen Konzept, das insbesondere psychische Erkrankungen auf umfassende, erbliche, fortschreitende „Entartungsprozesse“ zurückführte [16]. Dem gegenüber stand das frühere Konzept der Monomanien, einem von Jean Étienne Dominique Esquirol entwickelten Verständnis psychischer Erkrankungen auf der Grundlage der „manie sans délire“ [3], wonach eine Vielzahl krankhafter Verhaltensweisen langfristig nicht zur Beeinträchtigung der Verstandesfunktion führe. Benannt wurde die jeweilige Form der Monomanie nach der augenscheinlich vordergründigen Pathologie, dem sog. „Einzel-“ oder „Partialwahn“. Einzelne der ursprünglich als Monomanie bezeichneten Triebstörungen werden noch immer so bezeichnet, wie die Pyromanie, Kleptomanie, Nymphomanie oder Erotomanie. Auch oder gerade weil bei den Monomanien der terminologische Ursprung zu finden ist, will Kraepelin anhand des „impulsiven Irreseins“ das Konzept der solitären Monomanien zugunsten seiner Degenerationstheorie widerlegen. Konkret schreibt er, es liege „dasselbe Verhältnis vor uns wie bei manchen anderen Resten urwüchsiger Anlagen, wie namentlich bei der Hysterie und […] der Homosexualität und der Gesellschaftsfeindschaft“ ([23], IV. Bd., S. 1915), womit eindeutig sog. Entartungszeichen erkennbar seien. Zu einer ganz ähnlichen Einschätzung kam Richard von Krafft-Ebing in seinem *Lehrbuch der Psychiatrie* bereits im Jahre 1879. Impulsive Akte seien Ausdruck einer pathologischen Hypererregbarkeit, die wiederum durch einen psychischen Degenerationszustand verursacht sei, der entweder erblich, wie Epilepsie oder hysterische Neurosen, oder aber auch durch beispielsweise Trunksucht oder Onanie erworben sein könne. Diese Akte hätten somit „das Material zum Aufbau der Irrlehre von den sogenannten Monomanieen geliefert“ [24, S. 72]. 1910 untersucht die Ärztin Elly Dinkelacker (später: Wittmann) 24 kriminelle Frauen, darunter größtenteils Brandstifterinnen und Diebinnen, auf sog. Entartungszeichen und wird bei den meisten von ihnen fündig, insbesondere in Form von Epilepsie und sog. „Schwachsinn“, wodurch sie die Degenerationstheorie bestätigt sieht [6].

Gleich mehreren Formen des „impulsiven Irreseins“ attestiert Kraepelin eine hysterische Störung als häufigen Auslöser und Komorbidität [23], was sich mit der Einschätzung anderer Nervenärzte wie Siegfried Placzek oder Richard von Krafft-Ebing deckte, die insbesondere die Kleptomanie als Symptom einer hysterischen Störung ansahen [28]. Das unkontrollierte Triebleben der Frau ist ein zentrales Charakteristikum des umfassenden, misogynen Krankheitsmodells Hysterie, das im 19. Jahrhundert als Modediagnose galt [38], weshalb die Geschichte der weiblichen Impulskontrollstörungen kaum ohne die jahrhundertelange theoretische Vorarbeit der Hysterielehre denkbar erscheint.

### Die aufstrebende forensische Psychiatrie

Eine von Kraepelins frühesten Veröffentlichungen war sein aufsehenerregender Aufsatz *Die Abschaffung des Strafmaßes* von 1880 [4, 22], der eine Debatte über Sinn und Zweck von Gefängnisstrafen anfachte [29, 34]. Kraepelin forderte, dass Gerichte nicht mehr das Vergeltungsbedürfnis, sondern Resozialisierungsbestrebungen und den Schutz der Gesellschaft zur Grundlage ihrer Urteilsverkündung machen sollten. Kraepelins konkrete Äußerungen zur Auswirkung von Strafen auf die Prognose des „impulsiven Irreseins“ können daher als Stellungnahme in diesem psychiatrisch-forensischen Diskurs gewertet werden.

Da nach seiner Bestrebung die individuelle psychische Konstitution des Angeklagten berücksichtigt werden solle, war damit für die Festlegung des Strafmaßes nicht nur das Urteil der Juristen, sondern auch die Expertise der Psychiater in Gerichtsprozessen gefragt. Impulsstörungen waren für diese Entwicklung von großer Bedeutung, da hiernach auch Verbrechen ohne qualitative Bewusstseinsstörung aufgrund einer pathologischen Triebhandlung ausgeführt werden konnten, sodass die Beurteilung hinsichtlich Schuldfähigkeit und Strafe äußerst komplex war. Die von Ernst Platner 1797 postulierte „Amentia occulta“ hatte für den deutschsprachigen Raum den Auftakt zu einer Reihe von Diagnosen gebildet, die einen nicht ins Auge fallenden, nur Einzelaspekte der gesamten Person ergreifenden „versteckten Wahn“ beschrieben. Sie war ein wichtiger Markstein auf dem Weg hin zur Etablierung einer forensischen Psychiatrie [11]. Im Einbezug eines in den Gerichten wirkenden psychiatrischen Gutachters identifiziert David W. Jones sogar einen wesentlichen Beitrag für die Konstitution des bis dato randständigen jungen psychiatrischen Fachbereiches als angesehene Wissenschaft überhaupt [19]. Statt der universellen moralischen Sicht auf Verbrechen wurde die Person und der psychische Zustand des Täters oder der Täterin zum zentralen Gegenstand der Betrachtung.

## Kriminalität als pathologische Weiblichkeit

Die kriminelle Frau wurde im Laufe des 19. Jahrhunderts zu einer Schlüsselfigur in der Schuldfähigkeitsdebatte [37]. Kindsmord, Diebstahl oder Giftmischerei galten, ebenso wie Brandstiftung, als Verbrechen, die hauptsächlich von Frauen ausgeübt wurden [26, 37]. Die Anerkennung einer aus freiem Willen aggressiv, kaltblütig oder antisozial handelnden Täterin stellt die Geschlechterhierarchie infrage, da die kriminelle Akteurin als Subjekt entgegen den ihr zugeschriebenen weiblichen Charakteristika handelt [10, 37]. Das Zugrundelegen einer Psychopathologie diente als Erklärung für dieses Dilemma, zudem – so auch nach Kraepelin – ohnehin „das Weib mit seiner zarteren Veranlagung, mit der geringeren Ausbildung des Verstandes und dem stärkeren Hervortreten des Gefühlslebens weniger Widerstandsfähigkeit gegen die körperlichen und psychischen Ursachen des Irreseins besitzt“ ([23], I. Bd., S. 150) und somit für das Auftreten psychischer Erkrankungen prädisponiert sei. Das Klischee der geisteskranken, willensschwachen Delinquentin wurde dabei gerne von weiblichen Straftäterinnen selbst bedient, die sich vor Gericht eine Strafmilderung versprachen und damit auch häufig Erfolg hatten [38].

Das von Karsten Uhl und Melanie Grütter beschriebene Phänomen, kriminellen Frauen würde eine Abweichung von normativer Weiblichkeit zugeschrieben, welche sich in einerseits mangelnden weiblichen Eigenschaften, andererseits in einer Überformung derselben äußern könne [10, 37], lässt sich auch beim „impulsiven Irresein“ nachvollziehen. Die Schuldenmacherin erfüllt mit ihrer naiven, bescheidenen Art und ihrem fehlenden Gespür für Finanzen das Klischee der schutzlosen, gutmütigen und wenig verstandesbegabten Frau, woraus ihre Pathologie erklärbar wird. Brandstifterinnen und Kindstöterinnen gelten als charakterlich unreif, also als entwicklungsbedingt nicht zur erwachsenen Frau geworden. Der Giftmischerin wird hingegen eine berechnende Performation idealer Weiblichkeit unterstellt, da sie sich nach außen fürsorglich und liebevoll gebe, was wiederum alte patriarchale Ängste der hinterlistigen, gefühlskalten Rächerin bedient.

## Frauenkörper und Verbrechen

Derweil gewinnt der Körper für die Kriminalpsychologie wie auch für die Degenerationslehre durch anthropologische Einflüsse im späten 19. Jahrhundert weiter an Bedeutung. Der Vorreiter dieser Bewegung, der italienische Arzt und Kriminalanthropologe Cesare Lombroso und sein Schwiegersohn, der Soziologe Guglielmo Ferrero, beschäftigten sich in ihrer Monografie *Biologie und Psychologie der Verbrecherinnen und Prostituirten* aus dem Jahre 1894 explizit auch mit phänotypischen Charakteristika und dem Menstruationszyklus weiblicher Verbrecherinnen [26]. In 1885 und 1888 verfassten Rezensionen, in denen Kraepelin Lombrosos Erkenntnisse zum somatischen Habitus verschiedener Verbrechertypen auch im Hinblick auf die juristische Praxis untersucht, würdigt er dessen herausragenden Beitrag zur Begründung der methodisch-naturwissenschaftlichen Kriminalpsychologie [4, 35], wenngleich er als Verfechter einer zentralorganischen Ursachenforschung psychischer Erkrankungen und aufgrund seiner praktisch-klinischen Erfahrung der These körperlicher Stigmata als Degenerationszeichen immer weniger zustimmte [16, 35].

In der Anwendung auf den Frauenkörper bleibt Kraepelin widersprüchlich. Einerseits kritisiert er kastrierende gynäkologische Eingriffe aus psychiatrischer Indikation mangels Therapieerfolg, zudem, weil wie bei der Hysterie eine psychologische Komponente die wesentliche für die Entstehung der Erkrankung sei, andererseits erkennt er in somatischen gynäkologischen Erkrankungen eine Prädisposition für Formen des Entartungsirreseins [23, 34]. Auch Menstruationsstörungen schreibt er eine erhebliche Bedeutung für die Entwicklung psychischer Erkrankungen zu, womit er sich dem wissenschaftlichen Tenor früherer namhafter deutschsprachiger Psychiater anschließt [12, 24, 27, 30].

Der Glaube an den engen Zusammenhang von Menstruation und Krankheit ist historisch tief verwurzelt [8]. Für die Brandstiftungstendenz menstruierender Frauen hatte die Medizin sogar eine unmittelbare humoralpathologische Erklärung beigebracht. Durch Ansammlung von Blut im Körper kurz vor der Menstruation werde die Netzhaut verdunkelt, was den Reiz nach Licht und Feuer auslöse [37]. Zudem galt die Konstitution der Frau laut Viersäftelehre als kalt und feucht, welche sie in der Pubertät durch sengende Hitze auszugleichen suche [8].

Ähnlich historisch verankert ist die Verbindung der Frau zum Giftmord. Die Expertise über die frühe Pharmakotoxikologie, das Kochen und Extrahieren von Heilmitteln und Giften aus pflanzlichen Zutaten, war eindeutig weiblich besetzt [7, 8]. Zudem gilt das Gift als die Waffe der Unterdrückten. Frauen sind meist körperlich unterlegen, treten seltener in offene körperliche, brutale Konflikte und es wird ihnen daher herkömmlich unterstellt, beim Morden bedachter vorzugehen, wie beispielsweise beim Giftmord [37]. Lässt sich in Kraepelins Beschreibungen anderer Formen des „impulsiven Irreseins“ zumindest grundlegend ein Hinweis auf den sozialen Nährboden der Krankheit und ein gewisses empathisches Verständnis für die Frauen erkennen, so geht er mit der Giftmischerin ungleich härter ins Gericht. Kraepelins Ansicht, dass es sich dabei auch um einen triebhaften Akt handeln könne, ist eine Rarität und lässt sich sowohl in früherer als auch späterer psychiatrischer Literatur nicht nachvollziehen. Seine These stützt er durch die namentliche Nennung berühmter weiblicher Giftmischerinnen als Beweis für das Vorhandensein dieser Triebstörung. Einige dieser historischen Figuren untersuchte Erika Eikermann, die allerdings bei fast allen eben jene Motive herausarbeitete, die laut Kraepelin genau gegen das Vorliegen eines triebhaften Aktes sprechen würden, wie die Absicht finanzieller Bereicherung oder die Beseitigung einer unliebsamen Person, so im Falle der Marquise de Brinvilliers oder der Geheimrätin Ursinus [7].

## Resümee

Das „impulsive Irresein“ wird als Triebstörung von Emil Kraepelin in seine schlussendlich 1909 bis 1915 vorgelegte, umfassende Nosologie psychiatrischer Erkrankungen aufgenommen. Als wenig erforschte klinische Diagnose unterlag die Impulskontrollstörung in den letzten beiden Jahrhunderten einem stetigen Auffassungswandel durch die Psychiatrie. Das dargestellte Krankheitsbild berührte im 19. und beginnenden 20. Jahrhundert verschiedene wissenschaftliche Diskurse, gerade auch über Frauenbilder. Dafür steht Kraepelin prototypisch. Die von ihm beschriebenen Erscheinungsformen dieser Krankheit decken sich größtenteils mit ebenjenen durch Frauen verübten Straftaten, die im Verlauf des 19. Jahrhunderts eine umfassende Strafrechtsdebatte mitausgelöst und für die Entstehung der forensischen Psychiatrie eine immense Bedeutung hatten.

## Fazit für die Praxis

- Emil Kraepelin nimmt das Krankheitsbild „impulsives Irresein“ in seine wegweisende Nosologie psychischer Erkrankungen, die er in der 8. Auflage seines 1909 bis 1915 erschienenen Lehrbuches präsentiert, auf.
- Die von ihm beschriebenen häufigen Formen des „impulsiven Irreseins“ sind impulsives Brandstiften, Kindstöten, Stehlen, Giftmorden und Schuldenmachen. Damit entsprechen sie zu großen Teilen den als typisch weiblich angesehenen Straftaten um die Wende in das 20. Jahrhundert und sind mit verschiedenen Zuschreibungen pathologischer Weiblichkeit assoziiert.
- Anhand von Kraepelins Beschreibung des Krankheitsbildes lässt sich seine Positionierung zur Strafrechtsdebatte und Degenerationslehre nachvollziehen.
